# ZDOG: zooming in on dominating genes with mutations in cancer pathways

**DOI:** 10.1186/s12859-019-3326-z

**Published:** 2019-12-30

**Authors:** Rudi Alberts, Jinyu Chen, Louxin Zhang

**Affiliations:** Department of Mathematics and Computational Biology Programme, National University of Singapore, Singapore, 119076 Singapore

**Keywords:** Dominator tree, Cancer-causing genes, PI3K/AKT signalling, Cytoscape app

## Abstract

**Background:**

Inference of cancer-causing genes and their biological functions are crucial but challenging due to the heterogeneity of somatic mutations. The heterogeneity of somatic mutations reveals that only a handful of oncogenes mutate frequently and a number of cancer-causing genes mutate rarely.

**Results:**

We develop a Cytoscape app, named ZDOG, for visualization of the extent to which mutated genes may affect cancer pathways using the dominating tree model. The dominator tree model allows us to examine conveniently the positional importance of a gene in cancer signalling pathways. This tool facilitates the identification of mutated “master” regulators even with low mutation frequency in deregulated signalling pathways.

**Conclusions:**

We have presented a model for facilitating the examination of the extent to which mutation in a gene may affect downstream components in a signalling pathway through its positional information. The model is implemented in a user-friendly Cytoscape app which will be freely available upon publication.

**Availability:**

Together with a user manual, the ZDOG app is freely available at GitHub (https://github.com/rudi2013/ZDOG). It is also available in the Cytoscape app store (http://apps.cytoscape.org/apps/ZDOG) and users can easily install it using the Cytoscape App Manager.

## Background

Cancer is a disease caused by genomic mutations. A mutation in a proto-oncogene changes the gene into an oncogenic state in which the gene promotes uncontrolled cell proliferation, loses its function, or promotes the migration of tumour cells to form new tumours in other parts of the body [1]. With the advent of high-throughput genome sequencing technology, different genomic resources have become available for identifying cancer-causing mutations in oncogenes, including the Catalogue of Somatic Mutations in Cancer (COSMIC) [2] and The Cancer Genome Atlas (TCGA) [3].

The accumulation of cancer genomic data demonstrates mutational heterogeneity between different cancers and between different genomes of the same cancer. This reveals that only a handful of oncogenes mutate frequently, whereas many cancer-causing genes mutate rarely [3]. Better bioinformatics tools are therefore required for discovery of those cancer-causing genes with low mutation frequency, in addition to more genomic data. Cytoscape [4] is a popular platform for integrative analysis of omics data and visualization of biological networks. By taking advantage of this platform, we develop an app, called ZDOG, for visualizing to what extent mutations in a gene may affect a cancer signalling pathway using the dominator tree. It was first investigated by Lengauer and Tarjan [5] in program optimization and has been finding a variety of applications in ecology [6] and phylogenetic networks [7].

## Implementation

### The dominator tree model

**Fig. 1:**
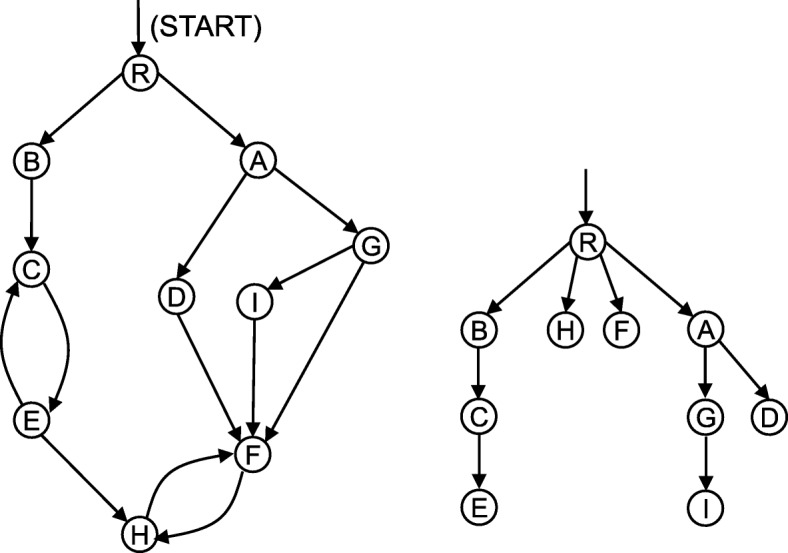
Illustration of the dominator tree model. A toy signalling pathway example (left) containing protein components A to I and R, where R is the signal entry point, and its dominator tree (right). Because of the right feedback loop between H and F, only the protein R completely controls F and H, indicating that a mutation occurring in R may affect the functions of F and H more than a mutation in any other component in the pathway

Consider a digraph *D* with a distinguished start node, called the root, in which every node is connected to the root by a path if arc orientation is ignored. We call a node *u* a dominator of another node *v* if every directed path from the root to *v* contains *u*. In the signalling pathway context, this implies that any signal flowing to the protein component *v* from the entry point must be relayed through the protein *u*. The dominator relation is a transitive binary relation on the vertices of *D*. It can be represented by a tree, called the dominator tree of *D*, in which there is an edge from *x* to *y* if *x* is the least dominator of *y* for every pair of nodes *x* and *y* of *D* [5]. The dominator tree is unique (Fig. 1, Additional file 1: Fig. S1) and computable in near-linear time [4].

### The ZDOG program

**Fig. 2:**
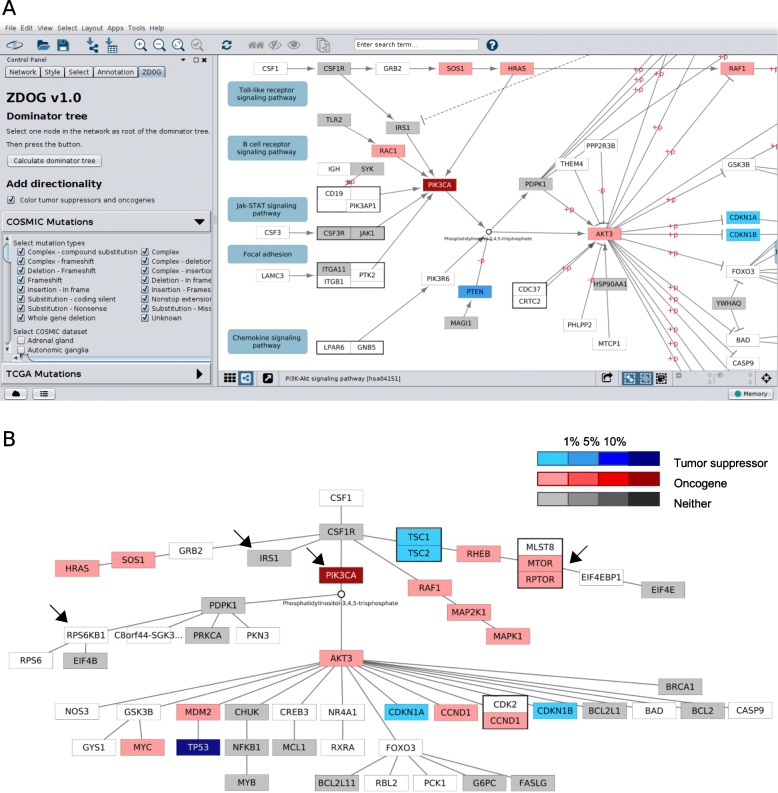
The graphical user interface and output of ZDOG. **a**. The interface window, where the user can select which mutation types and datasets available in either COSMIC or TCGA are used for analysis (left). The loaded pathway will be annotated with mutations and displayed. Mutated genes are colored red, blue or grey depending on whether they are oncogenes, tumor-suppressors or neither. Unmutated genes are not colored. By clicking the “calculate dominator tree” button, the user can examine the extent to which mutations in a gene will affect downstream protein components and signaling processes in the pathway under the dominator tree model. **b**. Dominator tree for the PI3K/Akt signaling pathway, with *CSF1* chosen as root. The genes are colored in terms of their mutation frequencies in the COSMIC breast cancer dataset. Protein complexes are represented by a box. Four additional dark arrows are used to highlight the key genes discussed in the case study

The ZDOG has two key functions (Fig. 2). First, it allows the user to examine gene mutations of different types in a cancer signalling pathway. After uploading a KEGG pathway from a local file or retrieving a pathway using KEGGscape [8], the user can examine genes that bear mutations in the datasets of the COSMIC and TCGA. Presently, the user can choose mutations of up to 16 types in up to 47 datasets available in the COSMIC and 18 types in up to 32 datasets in TCGA (Fig. 2a, left; Additional file 2: Table S1; Additional file 3: Table S2). Based on the selected mutation types and datasets, the mutation frequencies of the genes encoding protein components of the pathway are calculated and displayed in the right panel. Mutated genes are then coloured red, blue or grey according to whether they are oncogenes, tumour suppressors or neither, respectively, whereas unmutated genes are not coloured (Additional file 4). Additionally, the user can further examine mutations occurring in a particular gene by right-clicking the gene tag and then following “ZDOG – view mutation details” in the popped up context menu.

Second, the user can further zoom in on key mutated genes by viewing them in the dominator tree model of the signalling pathway (Fig. 2b). Here, we model a signalling pathway as a rooted connected digraph by selecting an entry point of the pathway signal, where each edge is oriented in the direction the signal flows in the pathway. Unfortunately, we are unable to distinguish inhibitory from stimulatory modification in the current implementation. The dominator tree is uniquely computed once the entry point of the signal is designated.

Re-examining mutated genes in the dominator tree of the signalling pathway allows the user to identify conveniently dominating mutated genes and to find out how they may affect the activation of downstream components that control important biological processes - cell proliferation, growth, apoptosis, etc. To this end, the user first selects a stimulus or a receptor protein as the entry point of the signal in the pathway displayed and then clicks the “calculate dominator tree” button. The gene mutations are redisplayed in the dominator tree of the pathway (Fig. 2b).

### Installation of ZDOG

As a Cytoscape app, ZDOG must be installed and run in Cytoscape 3.6.0 or later. Cytoscape is downloadable from http://www.cytoscape.org. ZDOG requires the yFiles Tree Layout procedure and the KEGGscape tool for support. After these programs are installed, ZDOG can be easily installed via the App Manager in Cytoscape. Simple search for ZDOG in the App Manager and click Install. Alternatively, the ZDOG-1.0.jar file can be downloaded from https://github.com/rudi2013/ZDOG and installed using the App Manager – install from local file. Presently, only pathways in KEGG format are supported. Detailed installation instructions can be found in the user manual (Additional file 5).

## Results

### A case study: Dysregulation of the PI3K/AKT pathway in breast cancer

The phosphatidylinositol 3-kinase (PI3K)/AKT signalling pathway (Fig. 2a; Additional file 1: Fig. S1) is one of the most frequently dysregulated signalling pathways in cancer [9]. Here, we use it to show how ZDOG can be used to infer key mutations in proto-oncogenes that may be responsible for the change in downstream biological processes.

With the colony stimulating factor 1 (CSF1) gene being chosen, the dominator tree of the PI3K/AKT signalling pathway is given in Fig. 2b. The structure of the dominator tree clearly suggests the following three basic facts about this signalling pathway:

- The key signalling subpath from PIK3CA to Phosphatidylinositol (3,4,5)-trisphosphate (PIP_3_) and to AKT (drawn in the middle of the tree) has full control on the downstream proteins that function in the glycolysis/gluconeogenesis process, the cell cycle process, the apoptosis process, the NF-kappa B signalling pathway and the p53 signalling pathway.
- The signalling pathway interacts with the erbB signalling pathway through the signalling subpath GRB2 to SOS1 and to HRAS. This subpath branches off from the key signalling subpath in (a) at the CSF1 receptor (CSF1R), suggesting that HRAS interacts with PIK3CA, but it cannot fully control PIK3CA and thus its downstream components. A similar observation can also be made for the signalling subpath RAF1 to MAP2K1 and to MAPK1 that interacts with the MAPK signalling pathway.
- In the dominator tree, the ribosomal protein S6 kinase beta-1 (RPS6KB1) is found in a path branching off at PIP_3_. This suggests that AKT is upstream of RPS6KB1, but cannot fully control it.

With complicated kinase interactions downstream of AKT, the PI3K/AKT signalling pathway contains a deep feedback loop in which RPS6KB1, which is downstream to the mTOR1 complex, directly inhibits insulin receptor substrate 1 (IRS1) [10]. The PI3K/AKT signalling pathway is mainly regulated through over-activation of proto-oncogenes such as *AKT*, subunits of PI3K and mTor through mutation and amplification, as well as loss of function of tumour suppressors such as PTEN. Because of the RPS6KB1 to IRS1 feedback interaction, over-activation of PI3K may occur through PTEN loss of function through this feedback besides through either upstream activation or the presence of activating mutations in PI3K itself. Therefore, therapy with either dual PI3K/mTOR inhibition or IGF-IR inhibition may provide an anti-proliferative advantage over specific AKT inhibitors [11]. This is consistent with the fact that the combination of both PI3K and mTor dominates proto-oncogenes of the pathway, but inhibiting AKT only does not fully control RPS6KB1 as shown in the dominator tree (Fig. 2b), fact (c) in the last paragraph. Moreover, Serra et al. [11] showed that cell lines harbouring *K-Ras* mutations were less sensitive to a dual PI3K/mTOR inhibitor NVP-BEZ235 than the rest of the tested cell lines. This corresponds well with the fact that neither PI3K nor mTor is a dominator of the Ras component in the signalling pathway (Fig. 2b), which is discussed in fact (b) listed above.

Overlaying mutations on the dominator tree of the PI3K/AKT signalling pathway clearly indicates that every downstream biological process was affected by some mutations occurring in breast cancer. In contrast, the MAPK signalling pathway was not affected much by mutation in breast cancer (Additional File 1: Fig. S2). This is consistent with the fact that PI3K/AKT is one of the major signalling pathways in breast cancer, but the MAPK signalling pathway is not [12].

## Discussion

The exploration of multidimensional cancer genomic data through visualization is a challenging task. Key visualization tools available for analysing cancer genomic data in the context of signalling pathways include PathwayMapper [13] and Cascade [14]. PathwayMapper can be used to overlay genomic alteration data from cBioPortal. It calculates and displays the frequencies of alterations in a signalling pathway. One of its nice features is distinguishing activating alteration from inactivation alteration through using positive and negative frequency. Cascade also allows multiple genomic alteration data to be simultaneously displayed onto a chosen biological pathway. It displays biological pathways together with alteration percentages of their protein components in a three-dimensional view.

Compared with PathwayMapper, Cascade and other similar visualization tools, ZDOG uses the dominator tree model to allow the user to examine the positional importance of a mutated protein component of a signalling pathway. For example, in the analysis of genome data in the case study (Fig. 2), the dominator tree (Fig. 2b) indicates that PIK3CA not only has a high mutation rate, but also is in a dominating position. On the other hand, EIF4B and PRKCA were mutated, but they are not in a dominating position. Hence, these two protein components likely carried passenger mutations.

## Conclusions

Because of its unique features, ZDOG allows biologists and clinicians to apply their domain knowledge to investigate dominating mutated components in a dysregulated signalling pathway. Such a model will be more demanded when new knowledge about hallmarks of cancer signalling pathways emerge, where more interactions among protein components are expected. In the future, we will also examine how to use the dominator tree for prioritization of cancer genes.

## Supplementary information

**Additional file 1: Fig. S1.** The complete phosphatidylinositol 3-kinase (PI3K)/Akt signalling pathway. **Fig. S2.** The KEGG MAKP signaling pathway and its dominator tree with TNF as the signaling entry point

**Additional file 2: Table S1**. Separation of 6,581,004 COSMIC variations into 47 organs/tissues

**Additional file 3: Table S2**. Datasets in the TCGA that are made available to ZDOG

**Additional file 4.** Supplemental File

**Additional file 5.** ZDOG user manual

## Data Availability

Project Name: ZDOG. Project home page: http://apps.cytoscape.org/apps/ZDOG Operating system(s): Platform independent. Programming language: Cytoscape plugin developed in Java. Other requirements: Java 1.6 or higher and Cytoscape 3.6.1 or higher. Licence: GNU GPL v3. Any restrictions to use by non-academics: no.

## Abbreviations

COSMIC: Catalogue of Somatic Mutations in Cancer
KEGG: Kyoto Encyclopedia of Genes and Genomes
TCGA: The Cancer Genome Atlas
