# Supplementary material for: ZDOG: zooming in on dominating genes with mutations in cancer pathways
Source: BMC Bioinformatics. 2019 Dec 30;20:740. doi: 10.1186/s12859-019-3326-z (PMC6937862; doi:10.1186/s12859-019-3326-z)
Supplement: Supplementary file 1 — Additional file 1: Fig. S1. The complete phosphatidylinositol 3-kinase (PI3K)/Akt signalling pathway. Fig. S2. The KEGG MAKP signaling pathway and its dominator tree with TNF as the signaling entry point [file 12859_2019_3326_MOESM1_ESM.pdf]

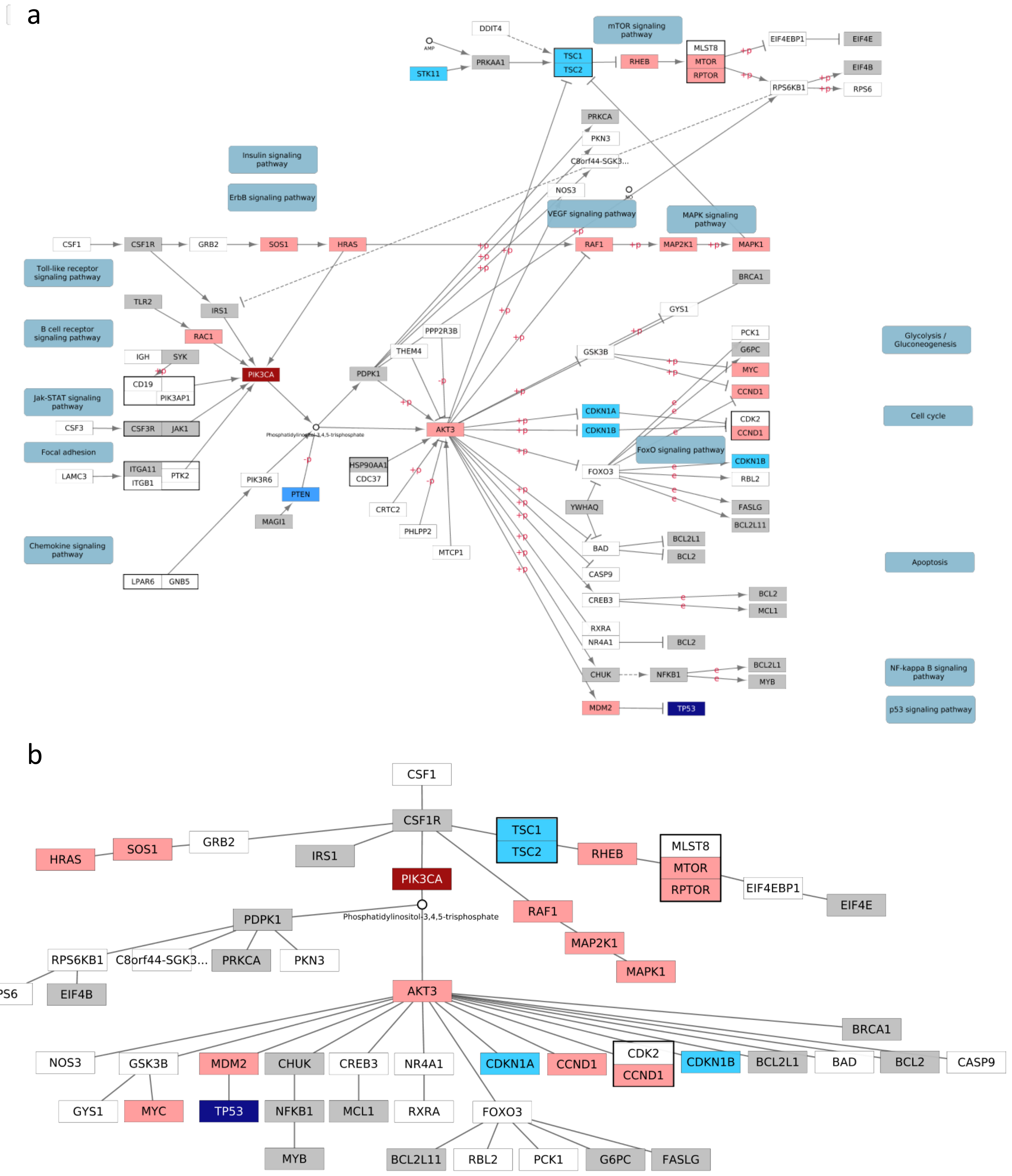

Figure S1. The complete phosphatidylinositol 3-kinase (PI3K)/Akt signalling pathway.
(a) It is modified from the KEGG PI3K/AKT signalling pathway (ID: hsa04151) by adding three feedback loop links reported in a survey paper by Dienstmann et al. (Molecular Cell Therapeutics, 2014, doi: 10.1158/1535-7163.MCT-13-0639).
(b) The dominator tree of the pathways when CSF1 is selected as the entry point of signal.

(a)

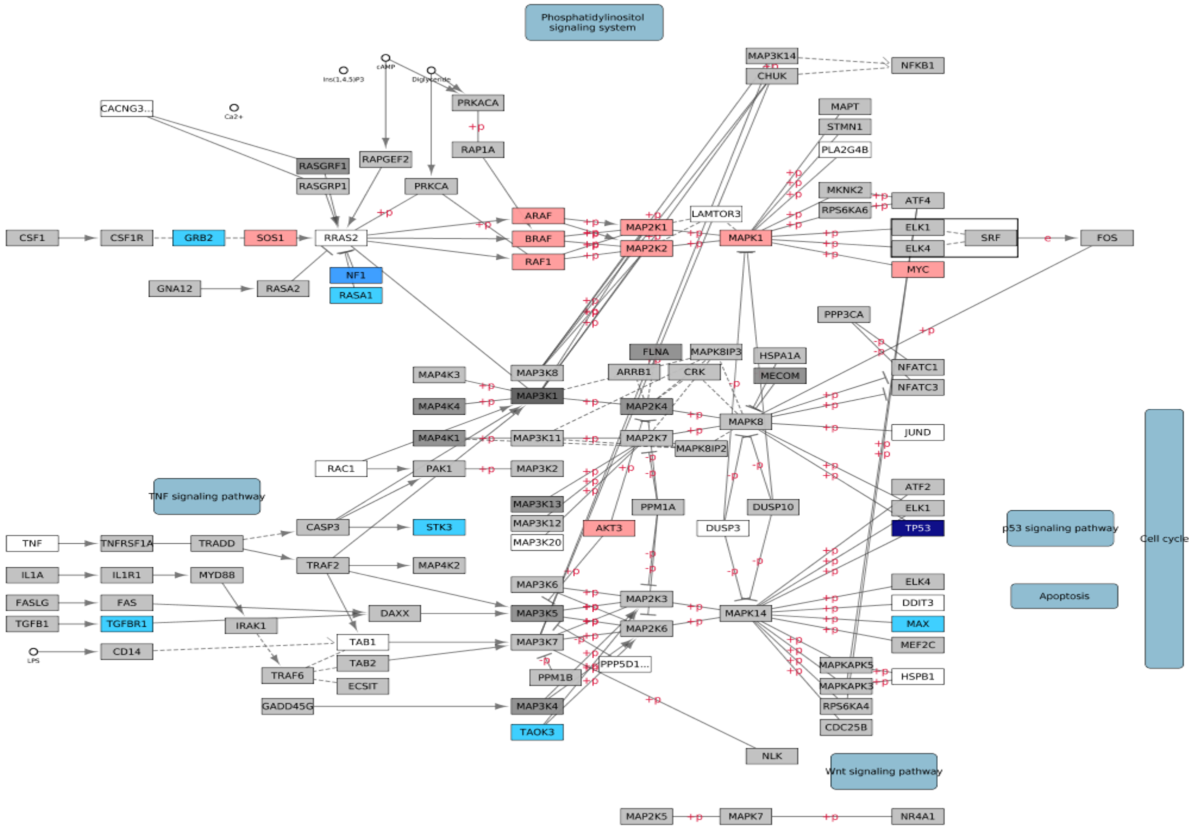

(b)

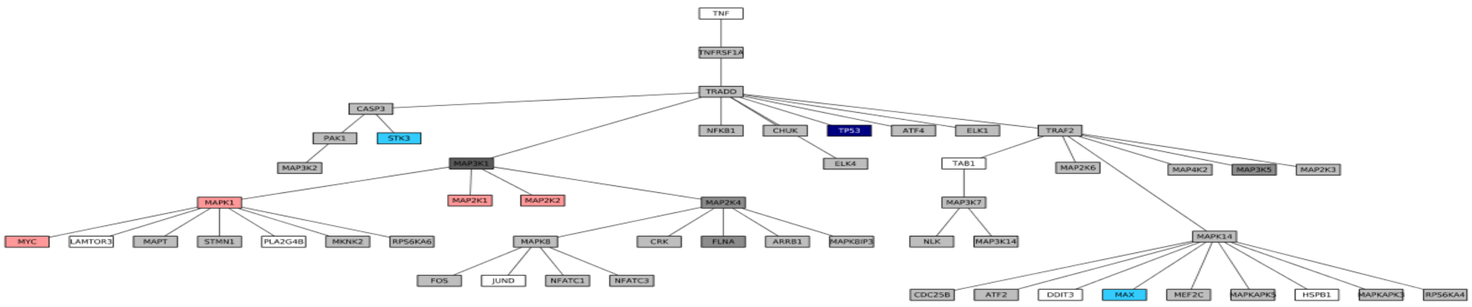

Figure S2. The KEGG MAKP signaling pathway and its dominator tree with TNF as the signaling entry point.
