# Supplementary material for: ZDOG: zooming in on dominating genes with mutations in cancer pathways
Source: BMC Bioinformatics. 2019 Dec 30;20:740. doi: 10.1186/s12859-019-3326-z (PMC6937862; doi:10.1186/s12859-019-3326-z)
Supplement: Supplementary file 2 — Additional file 2: Table S1. Separation of 6,581,004 COSMIC variations into 47 organs/tissues [file 12859_2019_3326_MOESM2_ESM.pdf]

Table S1: Separation of 6,581,004 COSMIC variations into 47 organs/tissues.

Number	Dataset	Number	Dataset
1	Adrenal gland	25	Paratesticular tissues
2	Autonomic ganglia	26	Parathyroid
3	Biliary tract	27	Penis
4	Bone	28	Pericardium
5	Breast	29	Perineum
6	Central nervous system	30	Peritoneum
7	Cervix	31	Pituitary
8	Endometrium	32	Placenta
9	Eye	33	Pleura
10	Fallopian tube	34	Prostate
11	Female genital tract	35	Retroperitoneum
12	Gastrointestinal tract	36	Salivary gland
13	Genital tract	37	Skin
14	Haematopoietic and lymphoid tissue	38	Small intestine
15	Kidney	39	Soft tissue
16	Large intestine	40	Stomach
17	Liver	41	Testis
18	Lung	42	Thymus
19	Mediastinum	43	Thyroid
20	Meninges	44	Upper aerodigestive tract
21	NS	45	Urinary tract
22	Oesophagus	46	Vagina
23	Ovary	47	Vulva
24	Pancreas		
