# Supplementary material for: ZDOG: zooming in on dominating genes with mutations in cancer pathways
Source: BMC Bioinformatics. 2019 Dec 30;20:740. doi: 10.1186/s12859-019-3326-z (PMC6937862; doi:10.1186/s12859-019-3326-z)
Supplement: Supplementary file 3 — Additional file 3: Table S2. Datasets in the TCGA that are made available to ZDOG [file 12859_2019_3326_MOESM3_ESM.pdf]

Table S2: Datasets in the TCGA that are made available to ZDOG.

Number	Dataset	Abbreviation
1	Adrenocortical Carcinoma	TCGA-ACC
2	Bladder Urotherial Carcinoma	TCGA-BLCA
3	Breast Invasive Carcinoma	TCGA-BRCA
4	Cervical Squamous Cell Carcinoma	TCGA-CESC
5	Cholangiocarcinoma	TCGA-CHOL
6	Colon Adenocarcinoma	TCGA-COAD
7	Lymphoid Neoplasm Diffuse Large B-cell Lymphoma	TCGA-DLBC
8	Esophageal Carcinoma	TCGA-ESCA
9	Blioblastoma Multiforme	TCGA-GBM
10	Head and Neck Squamous Cell Carcinoma	TCGA-HNSC
11	Kidney Chromophobe	TCGA-KICH
12	Kidney Renal Clear Cell Carcinoma	TCGA-KIRC
13	Kidney Renal Papillary Cell Carcinoma	TCGA-KIRP
14	Acute Myeloid Leukemia	TCGA-LAML
15	Brain Lower Grade Glioma	TCGA-LGG
16	Liver Hepatocellular Carcinoma	TCGA-LIHC
17	Lung Adenocarcinoma	TCGA-LUAD
18	Lung Squamous Cell Carcinoma	TCGA-LUSC
19	Mesothelioma	TCGA-MESO
20	Pancreatic Adenocarinoma	TCGA-PAAD
21	Pheochromocytoma and Paraganglioma	TCGA-PCPG
22	Prostate Adenocarcinoma	TCGA-PRAD
23	Rectum Adenocarcinoma	TCGA-READ
24	Sarcoma	TCGA-SARC
25	Skin Cutaneous Melanoma	TCGA-SKCM
26	Stomach Adenocarcinoma	TCGA-STAD
27	Thyroid Carcinoma	TCGA-THCA
28	Thymoma	TCGA-THYM
29	Uterine Corpus Endometrial Carcinoma	TCGA-UCEC
30	Uterine Carcinosarcoma	TCGA-UCS
31	Uveal Melanoma	TCGA-UVM
32	Ovarian Serous Cystadenocarcinoma	TCGA-OV
