# Supplementary material for: ZDOG: zooming in on dominating genes with mutations in cancer pathways
Source: BMC Bioinformatics. 2019 Dec 30;20:740. doi: 10.1186/s12859-019-3326-z (PMC6937862; doi:10.1186/s12859-019-3326-z)
Supplement: Supplementary file 4 — Additional file 4. Supplemental File [file 12859_2019_3326_MOESM4_ESM.pdf]

SUPPLEMENTARY FILE

ZDOG: Zooming in on Dominating Genes with Mutations in Cancer Pathways

Rudi Alberts, Jinyu Chen, Louxin Zhang

National University of Singapore, 10 Lower Kent Ridge Road, Singapore 119076

Table of Contents

Summary ... 3
Data sources and data preparation ... 4
Catalogue of Somatic Mutations in Cancer (COSMIC) ... 4
The Cancer Genome Atlas (TCGA) ... 4
Implementation ... 5
Colouring of allele frequencies for one dataset ... 5
Colouring of allele frequencies for multiple datasets ... 5
Separate colouring of tumour suppressors and oncogenes ... 6
Dominator tree algorithm ... 6
References ... 7

Summary

Inference of cancer-causing genes and their biological functions are crucial but challenging due to intertumoural and intratumoural heterogeneity of somatic mutation. We develop a Cytoscape app named ZDOG for visualizing the extent to which mutated genes affect a cancer pathway on the basis of the dominating tree concept. With just a few clicks, the user can view genetic variations in a biological pathway and examine the positional domination of a mutated gene in the pathway. This tool facilitates use of cancer genomics databases by enabling the identification of mutated "master" regulators in deregulated signalling pathways.

Data sources and data preparation

Catalogue of Somatic Mutations in Cancer (COSMIC)

Catalogue of Somatic Mutations in Cancer data was downloaded from the COSMIC website, COSMIC v87, released 13-NOV-18. Under the Data Downloads page (https://cancer.sanger.ac.uk/cosmic/download) section "COSMIC Mutation Data", we downloaded the tab separated table of 6,581,004 COSMIC coding point mutations from targeted and genome wide screens named

```
CosmicMutantExport.tsv.gz
```

We developed a bash script to extract the relevant columns from this file. Next, we wrote an R script to summarize mutation data per gene and per dataset. Additional file 1: Table S1 shows the division of the 6,581,004 variations into 47 datasets for different tissues / organs that we constructed using the "Primary site" column.

The Cancer Genome Atlas (TCGA)

The Cancer Genome Atlas data was downloaded from the NIH National Cancer Institute GDC Data Portal (https://portal.gdc.cancer.gov/). For each of 32 TCGA projects (Additional file 2: Table S2) we downloaded the open access Single Nucleotide Variation data file named like this:

```
TCGA.<dataset abbreviation>.mutect.*.somatic.maf.gz
```

We developed a bash script to extract the relevant columns from each of the files. Next, we wrote an R script to summarize the 3,172,730 variants per gene and per dataset.

Implementation

Using ZDOG the user can visualize genetic variations in either COSMIC or TCGA. For either one of these databases, the user can select the types of mutations he is interested in. Next, the user can select one or several datasets (within either COSMIC or TCGA) and average allele frequencies per gene are coloured on the pathway.

Colouring of allele frequencies for one dataset

Here, we describe how allele frequencies are calculated and coloured on genes. First, we check which mutation types are selected for the specific database (COSMIC or TCGA). Next, for each gene in the pathway, we collect all mutations for the selected mutation types. Next, for all selected mutations in the gene we collect the names of the samples that carry the alternative allele. Finally, we count how many *unique* samples are in this collection of names. We do this because the same sample can have the alternative allele for several mutations in the same gene. Now, let d be this amount of unique samples carrying alternative alleles, and let t be the total amount of samples in this dataset. Then, the allele frequency is calculated as:

$$100 \times \frac{d}{t}$$

The colour coding for the allele frequency is indicated in Figure 1.

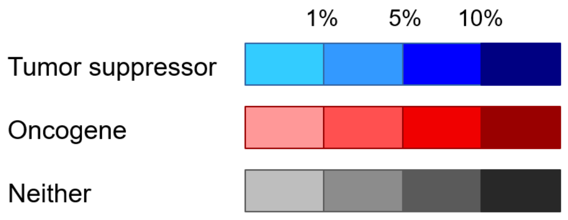

Figure 1 Colour coding for variation frequencies for tumour suppressors and oncogenes.

Colouring of allele frequencies for multiple datasets

Let d_i be the amount of unique samples carrying an alternative allele for selected mutation types per gene, as calculated in the previous paragraph, in dataset i. Let t_i be the total amount of samples in dataset i. The average allele frequency over multiple datasets N is simply calculated as

$$100 \times \sum_{i=1}^{N} d_i \; / \; \sum_{i=1}^{N} t_i$$

This average allele frequency is also colour coded using the coding in Figure 1.

Separate colouring of tumour suppressors and oncogenes

We took the collection of 187 tumour suppressors and oncogenes reported in Supplementary Table 4 of Sanchez-Vega *et al.*, 2018. We divided them into a list of 63 oncogenes and another list of 124 tumour suppressors. To color genes in ZDOG, we check whether they appear in one of those lists. If the gene is among the tumour suppressors, it gets a blue shade. If the gene is among the oncogenes, it gets a red shade. If the gene in not in the lists, it gets a grey shade.

Dominator tree algorithm

We implemented the fast algorithm for finding dominators in a flowgraph into ZDOG, originally introduced in 1979 (Lengauer and Tarjan, 1979). After selecting one (and only one) node in the pathway, the user can click "Calculate dominator tree" and the dominator tree will be presented in a new network window in Cytoscape. The previously selected node will be the root of the dominator tree. In this tree, dominating relationships between genes can be directly observed. Also, genes in the tree can be colour coded the same way as described above.

Since a gene can appear multiple times in a biological pathway, and since the dominator tree algorithm works on node names and also we are interested in the 'overall' dominating relations between genes, before running the dominator tree algorithm, we merge duplicate genes into one gene, i.e. each gene is represented by one node in the network.

Please be aware that a little rewiring of the pathway is sometimes necessary for complexes. It can be that a complex contains e.g. two genes and that genes in the complex are directly connected to upstream and/or downstream genes. In that case, edges of the genes in the complex should be rewired to the complex itself. This is because complexes are seen as nodes in the network, and the genes within the complexes not. In the case of KEGG pathways, all genes in complexes are "manually" copied back into the complex in the dominator tree, but the complexes themselves are nodes in the tree.

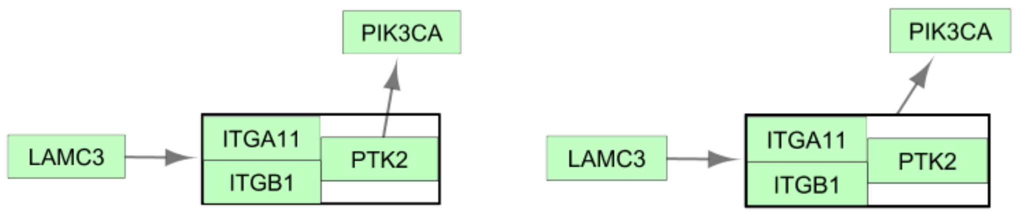

Figure 2. Example of rewiring of genes in complexes. Left: LAMC3 is connected to the complex but PTK2 is connected to PIK3CA. Right: LAMC3 is connected to the complex and the complex is connected to PIK3CA.

References

Lengauer,T. and Tarjan,R.E. (1979) A fast algorithm for finding dominators in a flowgraph. *ACM Trans. Program. Lang. Syst.*, **1**, 121–141.

Sanchez-Vega,F. *et al.* (2018) Oncogenic Signaling Pathways in The Cancer Genome Atlas. *Cell*, **173**, 321–337.
