# Supplementary material for: ZDOG: zooming in on dominating genes with mutations in cancer pathways
Source: BMC Bioinformatics. 2019 Dec 30;20:740. doi: 10.1186/s12859-019-3326-z (PMC6937862; doi:10.1186/s12859-019-3326-z)
Supplement: Supplementary file 5 — Additional file 5. ZDOG user manual [file 12859_2019_3326_MOESM5_ESM.pdf]

ZDOG: Zooming in on Dominating Genes with Mutations in Cancer Pathways

User manual v1.0.6

Contents

1 Cytoscape installation 2
2 yFiles Tree Layout and KEGGscape installation 3
3 ZDOG installation 6
4 Loading the PI3K-Akt and signalling pathways 8
5 Colouring mutations in COSMIC 11
6 Discriminating oncogenes and tumor suppressors 12
7 Colouring mutations in TCGA 13
8 Dominator tree calculation 14
9 Remarks on Protein Complexes 15

1 Cytoscape installation

ZDOG is an app developed for Cytoscape 3.7.1. ZDOG is known to run in Cytoscape versions 3.6.1 and 3.7.1. Cytoscape can be download from http://www.cytoscape.org. The interface of the program looks like this:

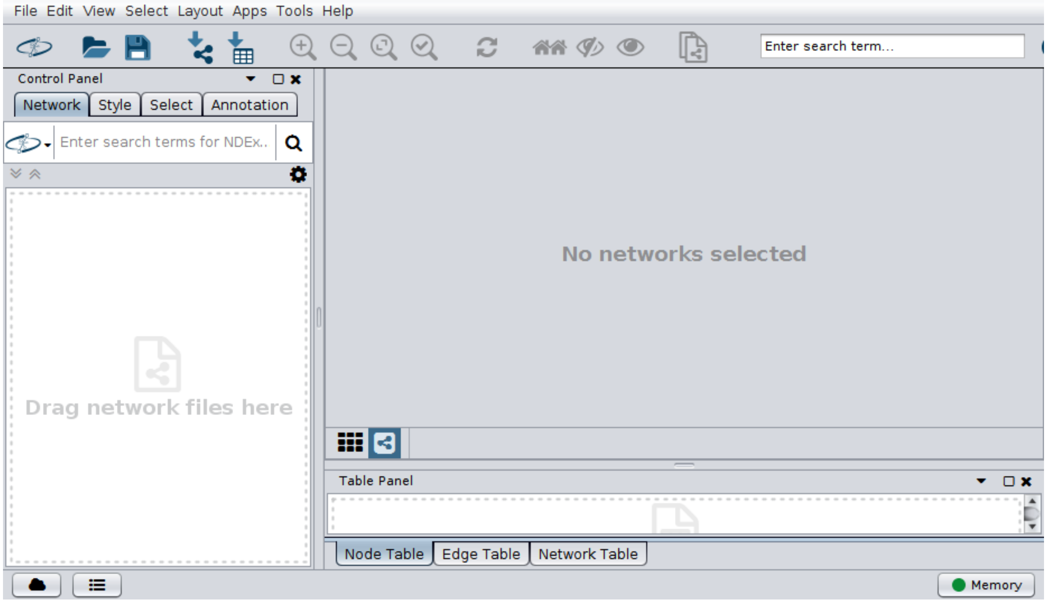

2 yFiles Tree Layout and KEGGscape installation

ZDOG makes use of yFiles Tree Layout and of KEGGscape. To install yFiles Tree Layout in Cytoscape, click on the “Layout” menu and then on “Install yFiles Tree Layout…”.

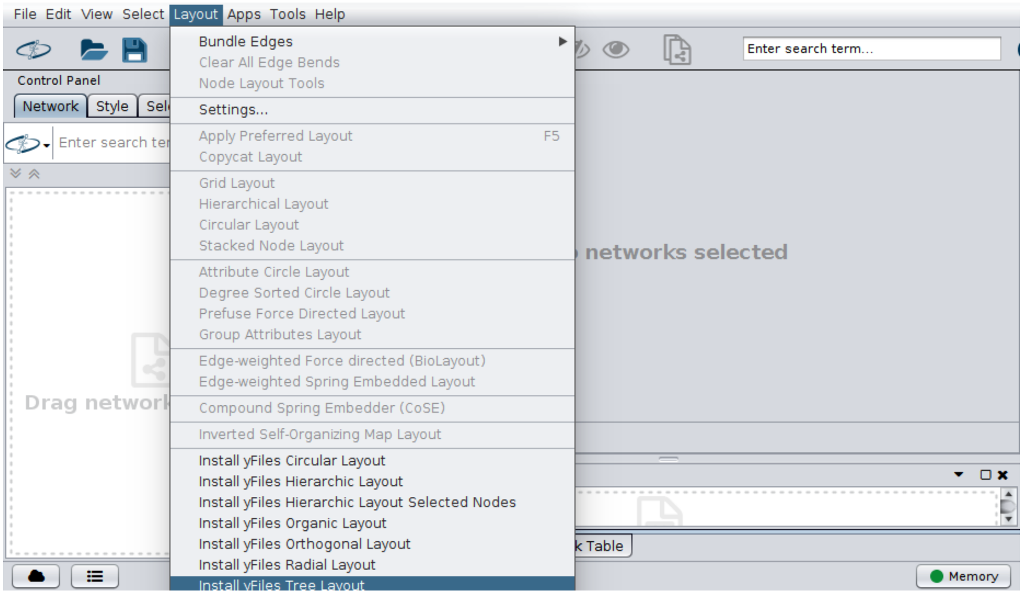

An internet browser such as Internet Explorer or Firefox will automatically open with the following webpage. On this webpage, click on the Download button on the right.

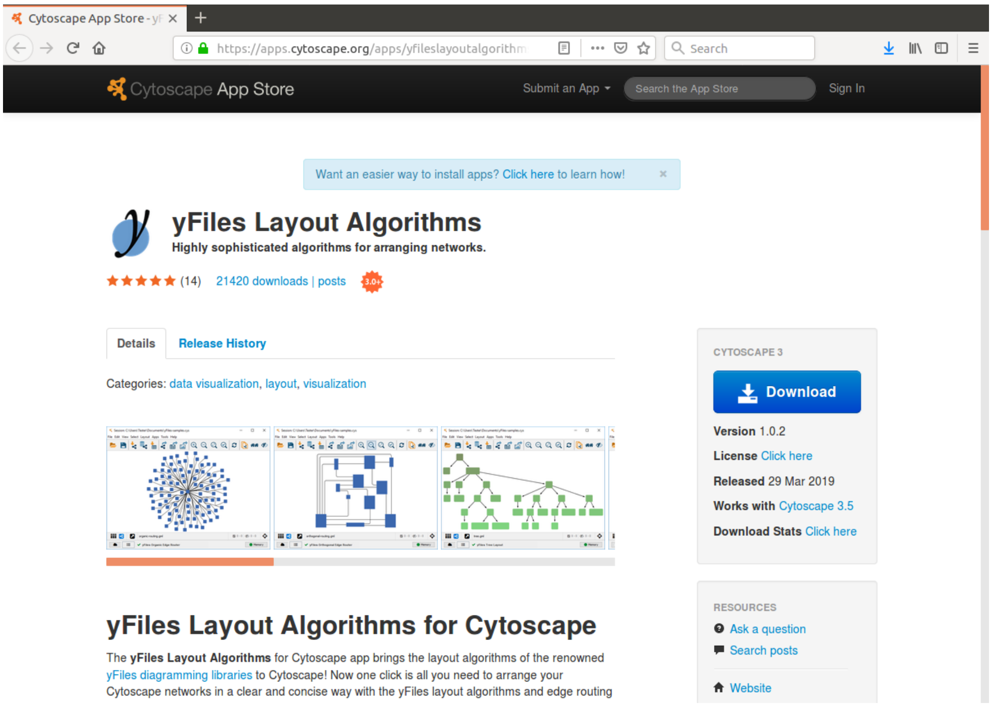

A file called yfiles-layout-algorithms.jar is now in your Downloads folder. Install this file in Cytoscape by clicking Apps – App Manager.. in the menu.

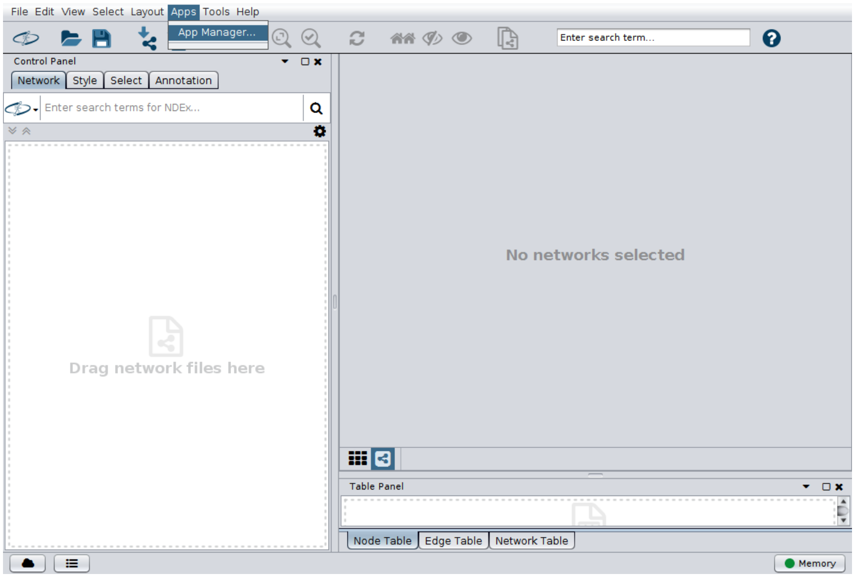

Click on the "Install from File…" button in the left bottom corner.

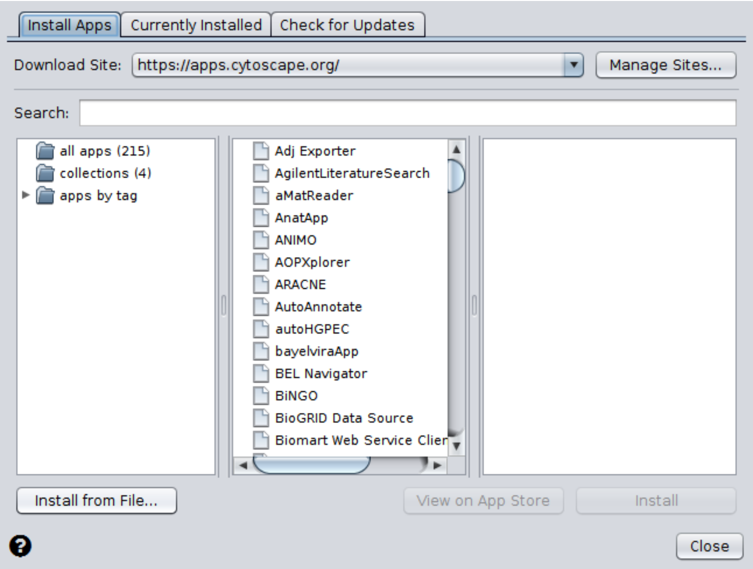

Select the file yfiles-layout-algorithms-version.jar and click "Open". After installation click "Close".

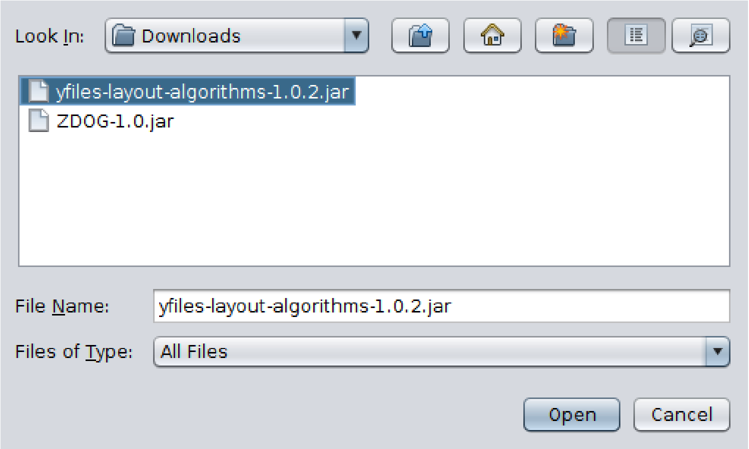

To install KEGGscape, open the App manager in Cytoscape.

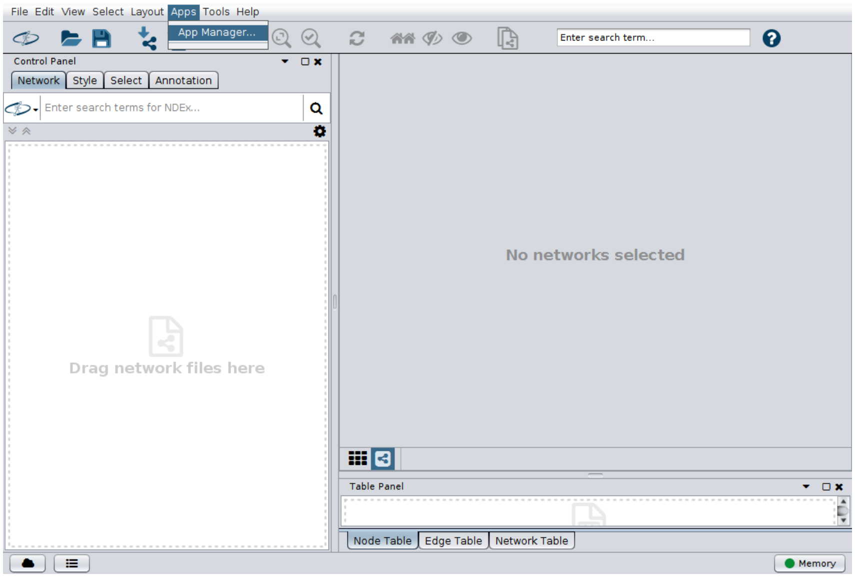

Next, search for "kegg", select KEGGscape and click the "Install" button. After installation, click "Close" to close the App Manager.

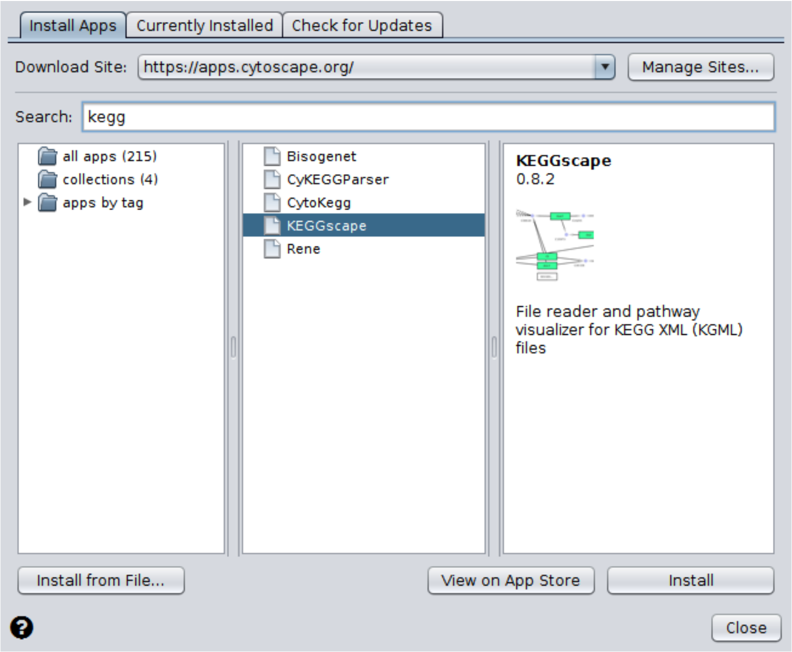

3 ZDOG installation

ZDOG can be installed via the App Manager of Cytoscape. In Cytoscape, click Apps – App Manager...

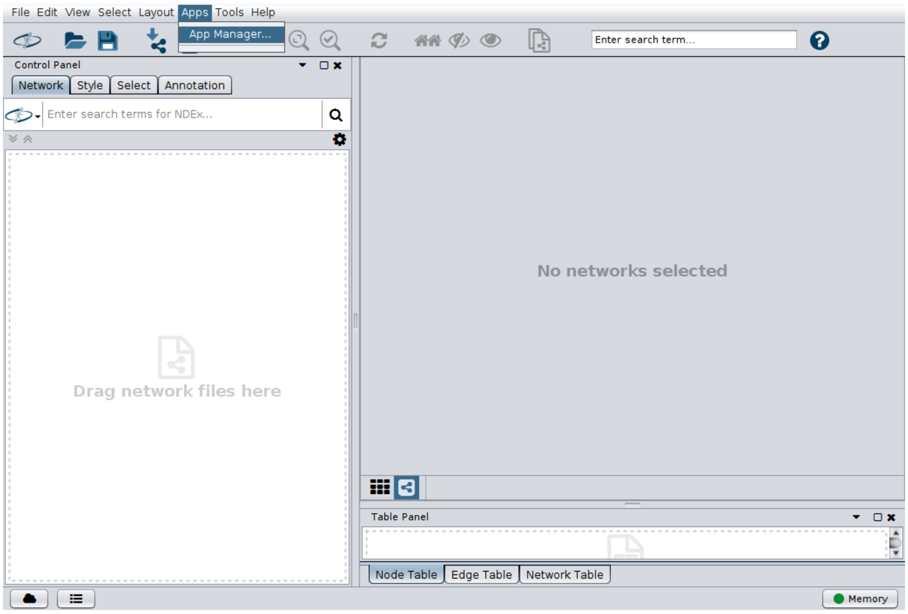

Scroll down to ZDOG, select it and click the "Install" button.

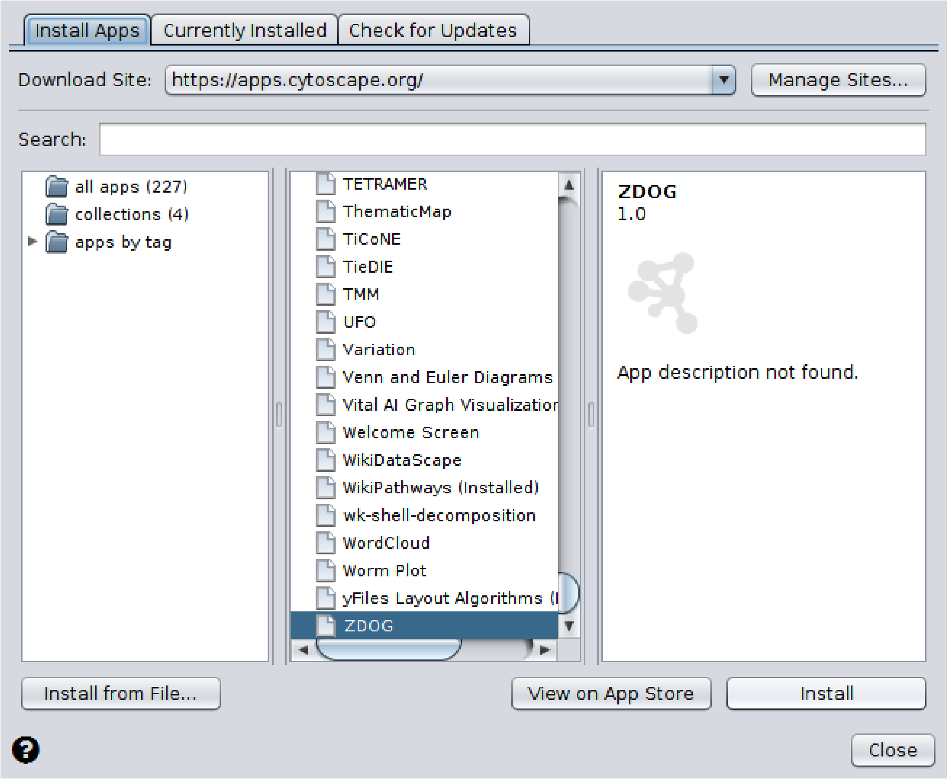

Once installed, the ZDOG menu is added to the Control Panel in Cytoscape on the left.

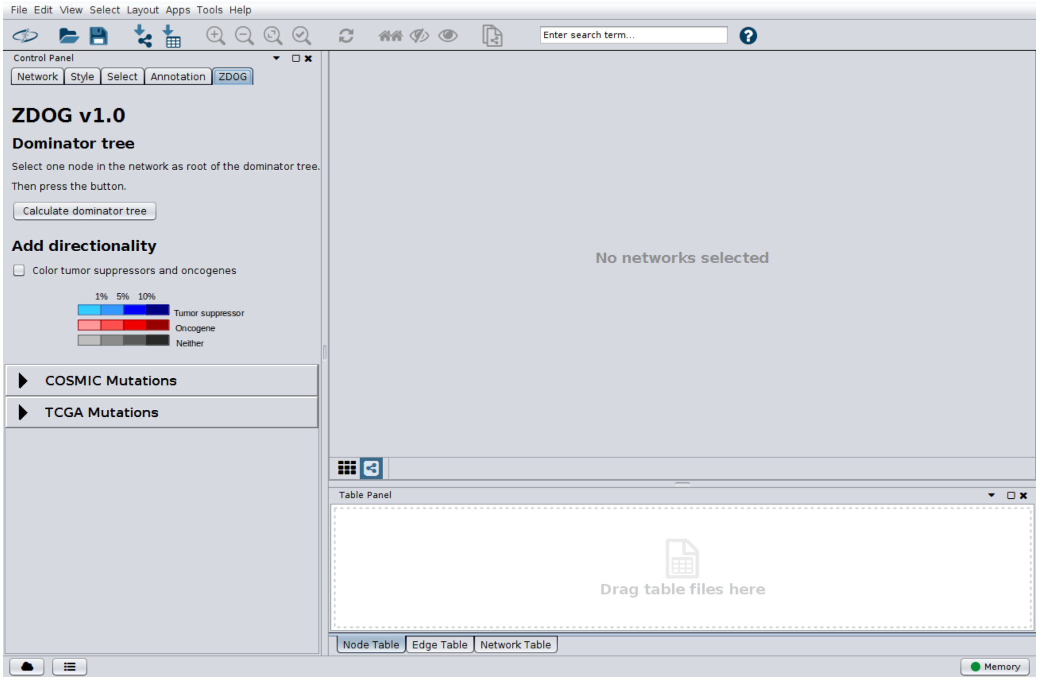

4 Loading the PI3K-Akt and signalling pathways

ZDOG is designed to work on KEGG pathways. The PI3K-Akt pathway that we used for case study can be downloaded as a Cytoscape 3 session file from GitHub and then opened in Cytoscape. Please browse here: http://github.com/rudi2013/ZDOG/blob/master/CytoScape3Session.cys and click the "Download" button on the website.

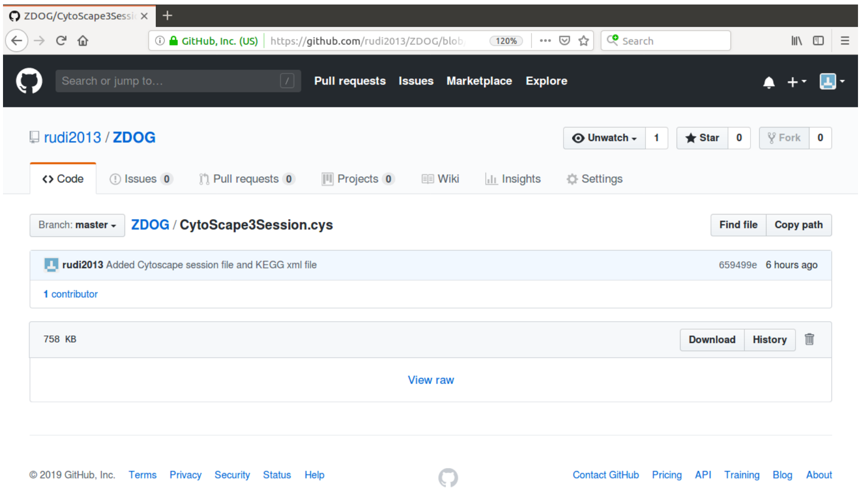

Alternatively, one can visit https://github.com/rudi2013/ZDOG and search the CytoScape3Session.cys file in the list. Click of the file and click the "Download" button on the website that opens. Once the session file is downloaded, start Cytoscape and click File – Open..

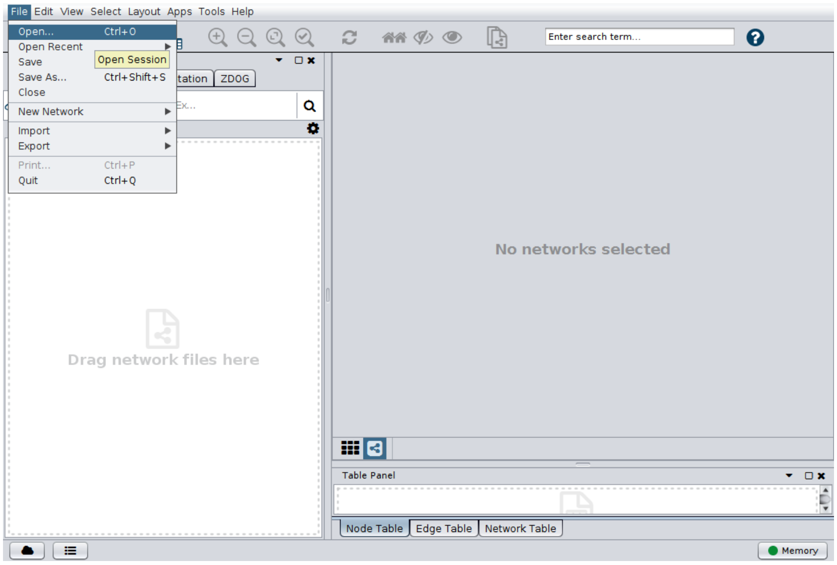

Then Browse to the CytoScape3Session.cys file, select it and click “Open”.

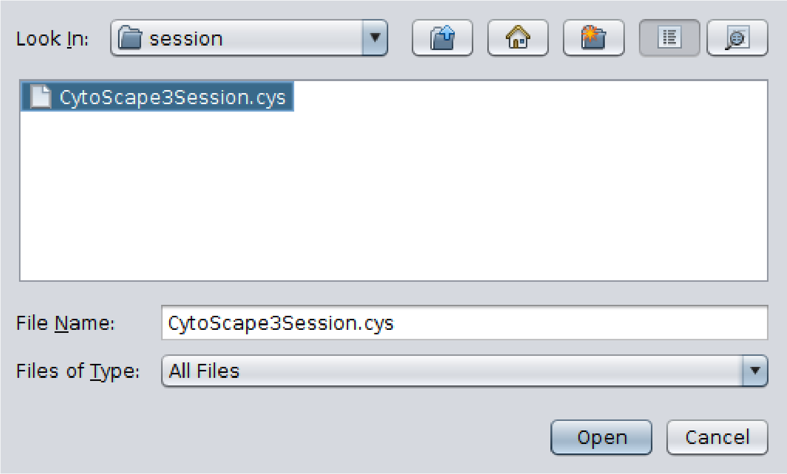

In general, other pathway files can be downloaded from the KEGG website. One can browse online to a KEGG PATHWAY and download the KGML file by clicking “Download KGML” above the pathway. For example, the homo sapiens PI3K/AKT pathway can be downloaded here: https://www.genome.jp/kegg-bin/show_pathway?org_name=hsa&mapno=04151

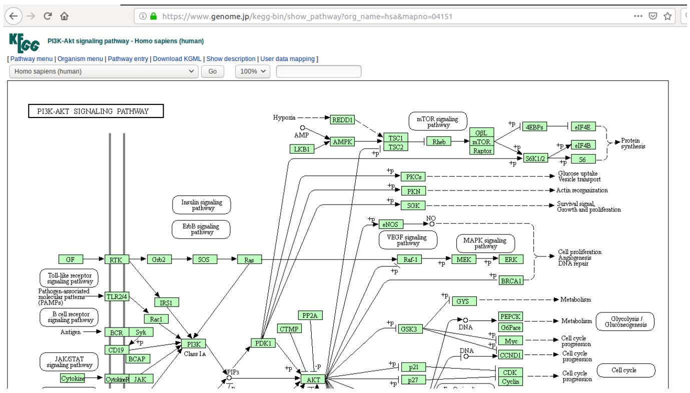

In Cytoscape, load the pathway by clicking File – Import – Network – File...

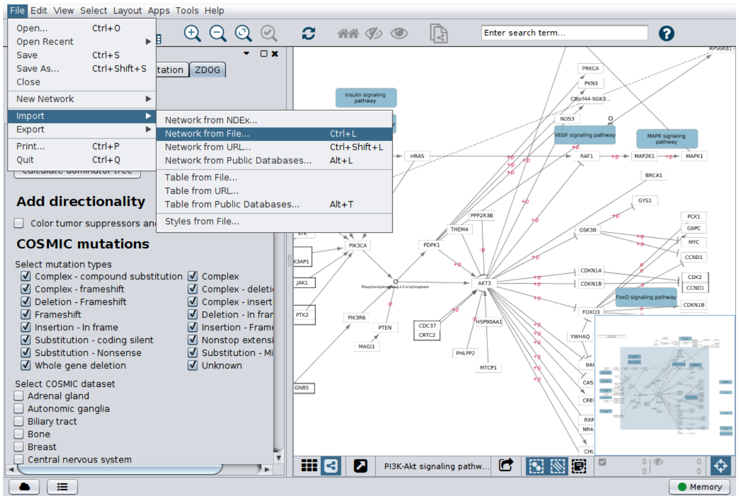

Browse to the downloaded KGML file (with extension .xml) and open it.

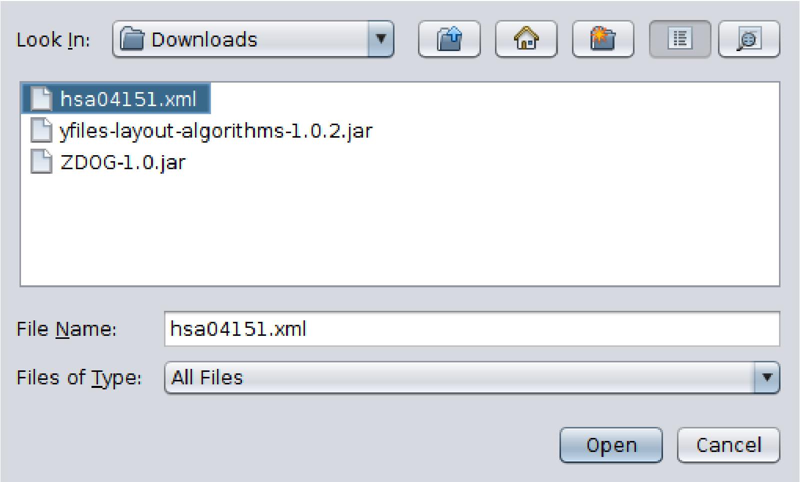

The original KEGG PI3K-Akt pathway file can also be found on the ZDOG GitHub page:
This is the GitHub page: https://github.com/rudi2013/ZDOG
This is the file: https://github.com/rudi2013/ZDOG/KEGG_PI3K_Akt_pathway.xml

Remark Only KEGG pathway files are supported by ZDOG. Please note that some dashed lines on KEGG Pathways on the KEGG website are not represented in the downloadable KGML files so they might be missing when the network is loaded in Cytoscape.

5 Colouring mutations in COSMIC

Under "COSMIC mutations" in ZDOG, select the wished mutation types and also one or several COSMIC datasets. Average mutation frequencies per gene will be instantly coloured on the pathway. All genes with mutations are coloured blue. Shades of blue indicate mutation frequency.

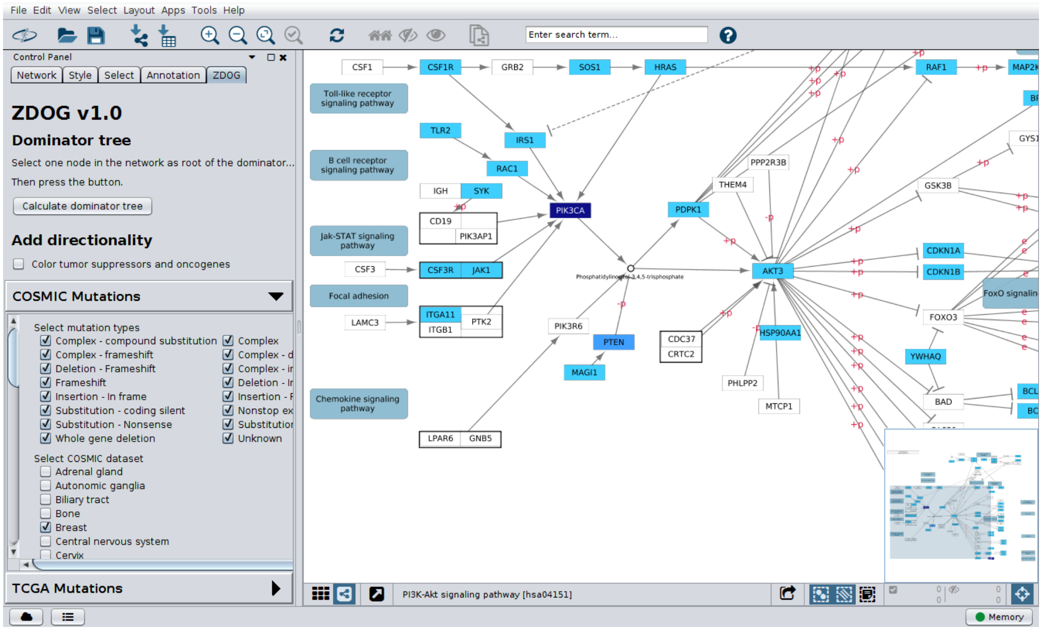

6 Discriminating oncogenes and tumor suppressors

Toggling the checkbox under “Add directionality” in ZDOG will colour oncogenes red and tumor suppressors blue and other genes grey.

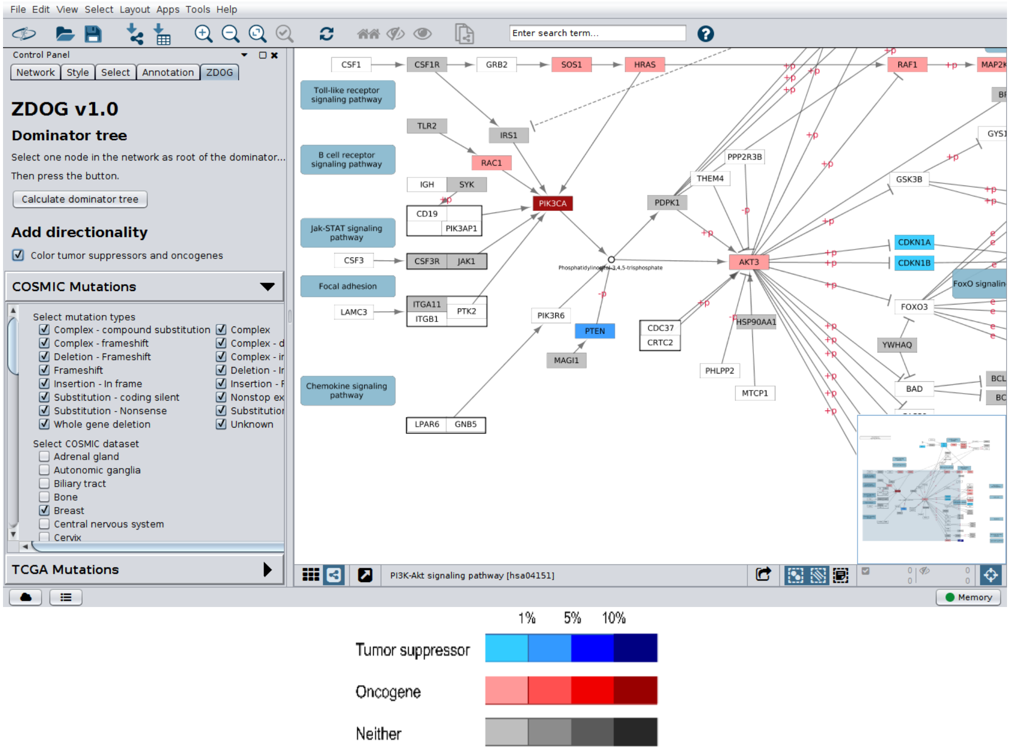

7 Colouring mutations in TCGA

Scroll down the scroll bar on the right of the graphical user interface of ZDOG until "TCGA mutations". Here, mutation types and TCGA datasets can be selected in the same fashion as above.

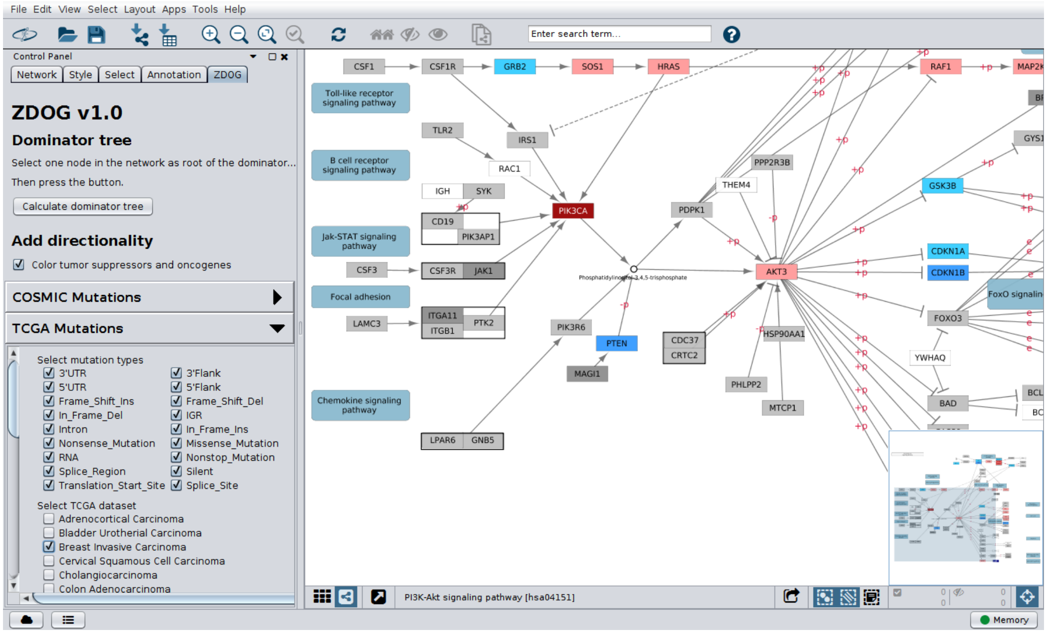

8 Dominator tree calculation

Select one and only one node in the loaded pathway. Here we selected "CSF1".

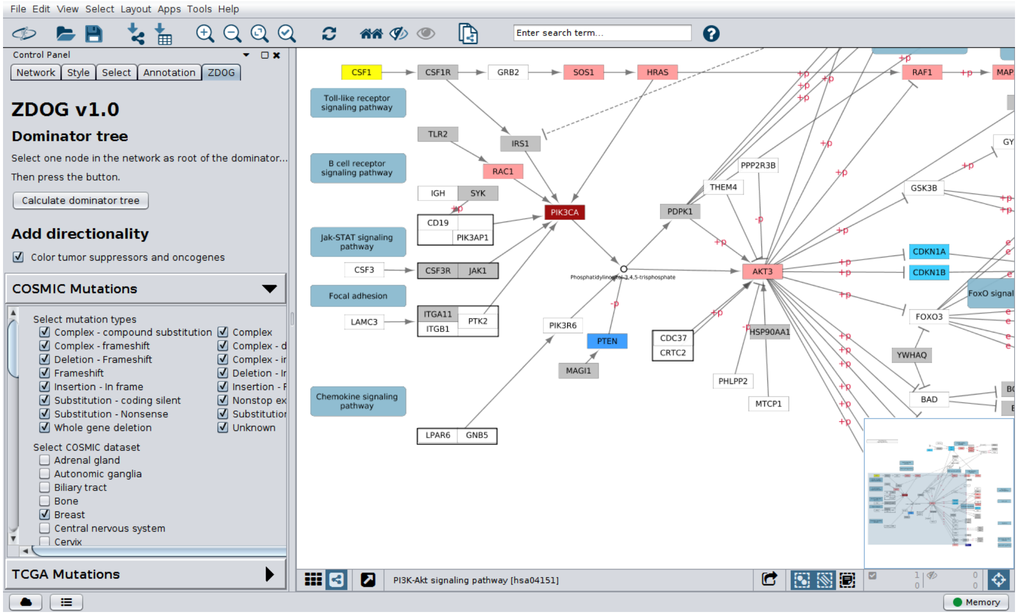

Click the "Calculate dominator tree" button to start the calculation. The tree will be presented in a new network window in Cytoscape (partially displayed).

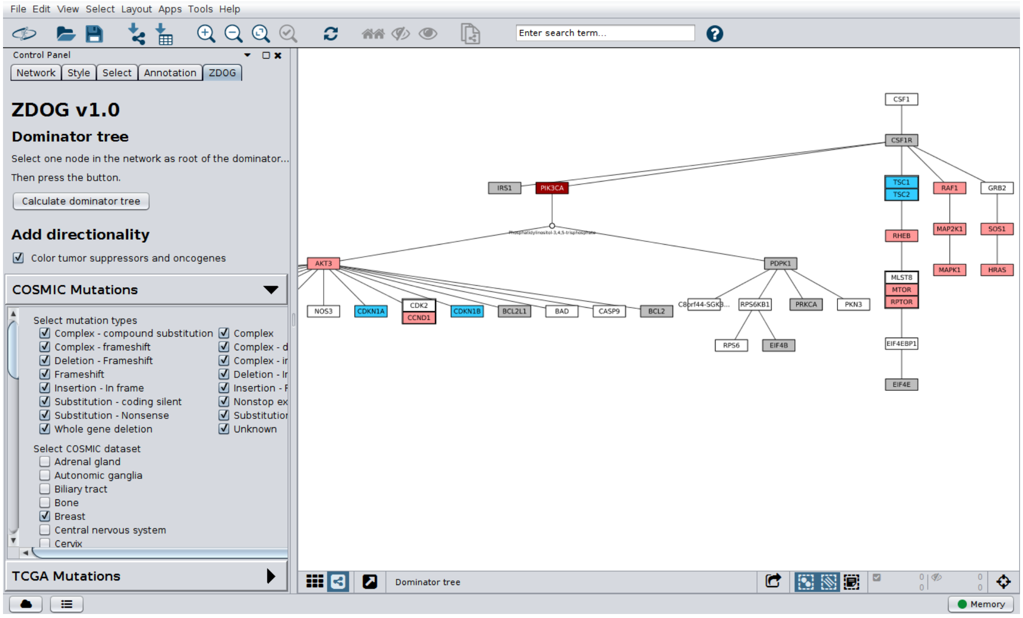

9 Remarks on Protein Complexes

For protein complexes, rewiring of the pathway needs sometimes to be done. The reason for this is that either proteins within complexes or the entire complex might be connected to up/downstream protein components. See the figure below for an example of rewiring genes in complexes. Left: LAMC3 is connected to the complex but the gene PTK2 is connected to PIK3CA. Right: LAMC3 is connected to the complex and PIK3CA is now connected to the complex and not anymore to the gene PTK2.

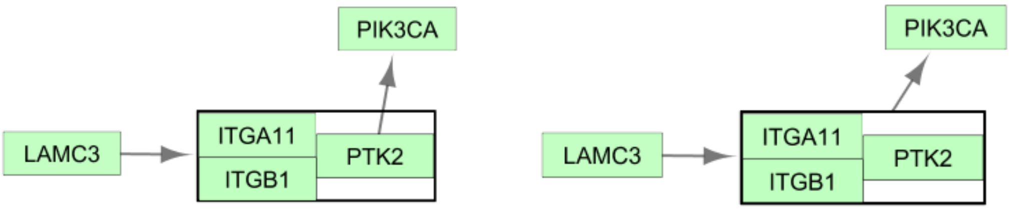

ZDOG treats the entire complex as a node in the network. For complexes to appear in the dominator tree, one has to make sure all incoming and outgoing edges are connected to the complex rather than individual protein members within the complex.

Remark   When a protein complex is selected to root the dominator tree, the complex is not colored yellow, unlike the case when a protein is selected.
